# Self-reported visual impairment and sarcopenia among older people in Cameroon

**DOI:** 10.1038/s41598-022-22563-9

**Published:** 2022-10-21

**Authors:** Salvatore Metanmo, Callixte Kuate-Tegueu, Antoine Gbessemehlan, Jean-Francois Dartigues, Marie-Josiane Ntsama, Laurella Nguegang Yonta, Andre-Pascal Kengne, Nadine Simo-Tabue, Maturin Tabue-Teguo

**Affiliations:** 1 Inserm U1094, IRD U270, Univ. Limoges, CHU Limoges, EpiMaCT-Epidemiology of Chronic Diseases in Tropical Zone, Institute of Epidemiology and Tropical Neurology, Omega Health, Limoges, France; 2 Neurology Department, Laquintinie Hospital of Douala, Douala, Cameroun; 3 Inserm U1219 Bordeaux Population Health Center, Université de Bordeaux, Bordeaux, France; 4 Faculty of Medicine and Biomedical Sciences, The University of Yaoundé 1, Yaounde, Cameroon; 5 Non-Communicable Disease Research Unit, South African Medical Research Council of South Africa, Cape Town, South Africa; 6 CHU de Guadeloupe, Equipe LAMIA, Université des Antilles, Fouillole, Guadeloupe

**Keywords:** Geriatrics, Epidemiology, Eye diseases

## Abstract

Aging has been clearly associated with decline in visual and physical performance. Alteration of visual function is associated with negative health outcomes including physical frailty. We assessed the relationship between Visual Impairment (VI) and sarcopenia in older persons in Cameroon. In a cross-sectional survey conducted in Douala in 2019, sarcopenia was assessed using the SPPB (Short Physical Performance Battery) test scored from 0 to 12. The diagnosis of sarcopenia was based on SPPB test score < 9 while VI was self-reported. Of the 403 participants (50.4% male) with a mean age of 67.1 (± 6.2) years, 356 (88.3%) reported a VI while the prevalence of sarcopenia was 47.9% [95% CI 43.0–52.7]. After adjusting for several factors, VI was significantly associated with sarcopenia (OR 2.66 [95% CI 1.29–5.48]). Of the SPPB subtests, only chair stand test was negatively associated with VI (β = − 0.45 [95% CI − 0.82 to 0.07]). Our study supports an association between VI and sarcopenia. If confirmed by further cohort studies, this result would suggest that VI could be considered as an early indicator of sarcopenia among older people in sub-Saharan Africa.

## Introduction

Physical impairments are common health issues with advancing age. Several older people experience these conditions which includes physical frailty and declining muscular mass and/or performance (i.e., sarcopenia)^1,2^. Current evidence shows that the presence of physical impairments (sarcopenia and/or physical frailty) is associated with multiple adverse conditions and increased risk of disabilities and untimely death in older people^3–9^. One of public health strategies to reduce these risks consists of identifying and controlling the modifiable determinants of physical well-being. It is well known that there is a link between visual impairment (VI) and undernutrition in older people^10^. Undernutrition is a risk factor for sarcopenia and physical frailty^11–13^ although all causes of these physical impairments are yet to be fully established^2,14–16^. Growing research supports that visually impaired people are more likely to have physical impairments^14,15,17–19^. These findings seem to suggest that VI could be an early determinant of the onset and/or worsening mechanisms of these physical impairments in older people.

VI is the commonest sensory impairment in sub-Saharan African (SSA) older people (e.g.: 69% prevalence in Congolese older people^20^); many of whom also experience VI consequences including physical impairments^19^. Knowledge on the association between VI and physical impairments in SSA older population is limited. The only study from SSA we are aware of, has reported an association of self-reported VI with frailty but not to pre-frailty status^19^. Physical frailty and sarcopenia may share the same determinants in addition to sarcopenia being a determinant of physical frailty^16^. Studies on the association between VI and sarcopenia in SSA are limited to a recently published study across six low- and middle-income countries-LMICS including Ghana and South Africa^21^. This study however did not report African-specific data. In the current study we first aimed to investigate the association between self-reported VI and sarcopenia among older people in Cameroon. Secondary, we explored the association between VI and physical frailty.

## Methods

### Study design and population

We conducted a general population-based cross-sectional study in the city of Douala in Cameroon from 1st January 2019 to 31 May 2019. The survey was among subscribers of the “Mutuelle des personnes âgées du Cameroun” (MUPAC). MUPAC is a non-political, non-profit humanitarian association created on April 2003, whose main objective is to negotiate the provision of health care to the older people. Aged 55 years and over was the only criterion to become member. MUPAC has about 2000 members in Douala. The executive committee of our project send an invitation to participate on all the members of MUPAC. A total of 615 volunteers agreed to participate. Among them, 403 aged 55 and above, who were able to stand up alone and walk four meters without assistance were eligible to participate. Older people with severe medical conditions (e.g.: respiratory pathology, congestive heart failure), severe physical pain/disability, neurological disease that can lead to cognitive impairment (epilepsy, systemic brain disease, recent stroke), and those with blindness were excluded. These serious medical conditions could compromise the diagnostic test performance.

### Survey procedures

MUPAC holds monthly meetings at its headquarters in each of the six districts of the city of Douala. Participants were recruited from 5 districts at MUPAC meetings between first January 2019 and 31th May 2019. At each MUPAC meetings, the study purpose was presented to the participants. Then, they were enrolled in the study if only they met all eligibility criteria and provide oral and written consent. Data were collected on (1) Socio-demographic characteristics: age, sex, occupation, education level, marital status; (2) Medical histories: hypertension, diabetes, chronic alcoholism, smoking; (3) Sarcopenia using the SPPB (Short Physical Performance Battery) that includes three subtests: balance test, Gait speed and 5-chair lift test. In addition, using the SOF index (Study of Osteoporotic Fractures also based on three indices: weight loss, 5-chair lift test and reduction in energy level) we collected data related to physical frailty. The assessment procedure for these tests is presented in the Appendices 1 and 2. (4) Finally, cognition status was assessed using the Mini Mental-State Examination. Other data collected included ADL (Activities of daily living), IADL (instrumental activities of daily living), depression (CES-D scale: Center for Epidemiologic Studies-Depression), falls and sensory impairment (vision and hearing). Participants were asked whether they had been diagnosed with a VI by an ophthalmologist. If the participants answered “Yes” to this question then the were considered as having a VI. We use a similar approach for hearing impairment and for the correction of these two sensory deficits.

### Measures

The outcome of interest was sarcopenia, which was assessed using SPPB, with a score ranging from 0 to 12. Participants with a score ≤ 9 were considered as having possible sarcopenia. The exposure of interest was presence or not of VI; based on self-reports. VI was defined as any decrease in visual acuity in a participant. If a participant reported having visual impairment, VI was coded as 'Yes'. Covariates used in regression models were age, sex, marital status, education, cognitive status, IADL score, nutritional status, hypertension, and diabetes. The Mini Nutritional Assessment (MNA) was used to explore nutritional status. The short version of the MNA was first used in all patients. Nutritional status was considered normal for all scores of 12–14 on the short version of the MNA. For all those with a score below 12 the long version of the MNA was used for further clarification. Participants with a score between 17 and 23.5 on the long version of the MNA were considered to be at risk of undernutrition.

### Statistical analysis

We first described all relevant variables according to age and sex with groups comparisons using appropriate tests. The associations between self-reported VI and sarcopenia were explored using logistic regression models. Two separate models were constructed: a basic model adjusted for age and sex and an expanded model adjusted for all covariates. Odds ratios (ORs) with the accompanying 95% confidence (95% CI) intervals were reported. Two secondary analyses were performed. In the first, the relationship between VI and physical frailty (assessed by the SOF index scale) was studied by categorizing SOF score into two groups: robust (score = 0) and frail (score ≥ 1). The second analysis investigated the association between VI and the different components of SPPB (considering each component as an outcome). These variables were modelled in their quantitative form. Each score ranged from zero to four. The associations were explored using multivariable linear regression models. The regression coefficients and accompanying 95% CI are reported.

Interactions between VI, age and sex were investigated in all models. Linearity of quantitative variables was checked using fractional polynomials. The normality and homoscedasticity of the standardized residuals were checked for the linear models. The software R version 4.0.3 was used for all statistical analyses.

### Ethical approval

This study received an ethical clearance (No. 2019/049/UdM/CIE) from the institutional ethics committee of the “Université des Montagnes” (Bangangté-Cameroon). We confirm that all experiments were performed in accordance with relevant guidelines and regulations. All the study participants provided written informed consent to participate in the study.

## Results

### Characteristics of the population by age and sex

Tables S1 and S1bis present the characteristics of the population by age and sex. The average age of the women was 66.7 years. Of the 200 women in our sample, the majority (51.5%) had between 65 and 75 years. The proportion of participants living alone increased with age, and was high (95.5%) in the oldest age group. The majority of women (53.5%) had attended at most secondary school. The women were on average obese and about 56% were still active. The prevalence of diabetes and hypertension increased with age. Cognitive impairment was more important in the oldest age group (95.5%). About 82% of the women, aged 75–88 years were frail. Many women reported VI (92.5%) (Table S1).

Men (n = 203) were on average 67.6 years old and, as women, those in the oldest age group were more likely to live alone (21.9%) than others. In all, 17.7% had reached higher education, with the highest proportion among the less old (26.2%). Only 25.1% of the men were still in employment and this percentage decreased with age. The prevalence of diabetes appeared to decrease with age while hypertension increased with age. Cognitive impairment, which affected 43.3% of the male population was more important in the older group (59.4%). The proportion of men with possible sarcopenia was 37.9% and increased with age (29.2% vs 35.8% vs 62.5%). The prevalence of vision and hearing impairments increased with age. Falls were reported by only 0.5% of men (Table S1 bis).

### Characteristics of the population according to the sarcopenia

The prevalence of sarcopenia was 47.9% [95% CI 43.0–52.7]. The distribution of age, sex, scolarity, cognitive impairment, IADL score, SOF index score and fall was statistically different between sarcopenia groups (Table 1). For instance, compared to women, men were less likely to have a sarcopenia (40% vs. 60%) while people living alone were more likely to have sarcopenia (51.8%) compared to those living in couples (36.5%). People with higher education level were less likely to have a sarcopenia (6.7%). Frequency of cognitive impairment was greater in people with sarcopenia. Compared to participants without sarcopenia, those with this condition had more VI (83.8% vs. 93.3%). BMI and undernutrition didn’t differ between sarcopenia groups.

**Table 1:** Sociodemographic and clinical characteristics of participants according to sarcopenia, Douala–Cameroon, 2019.

Characteristics	Overall, N = 403^a^	No sarcopenia, N = 210^a^	Possible sarcopenia, N = 193^a^	p value^b^
Age (years)	67.1 (± 6.2)	66.3 (± 5.6)	68.1 (± 6.7)	0.003
Sex (men)	203 (50.4%)	126 (60.0%)	77 (39.9%)	< 0.001
Marital status (lives alone)	176 (43.7%)	76 (36.2%)	100 (51.8%)	0.001
**Scolarity**				< 0.001
None/primary	125 (31.0%)	44 (21.0%)	81 (42.0%)	
Secondary	230 (57.1%)	131 (62.4%)	99 (51.3%)	
Higher	48 (11.9%)	35 (16.7%)	13 (6.7%)	
Being professionally active	163 (40.4%)	77 (36.7%)	86 (44.6%)	0.106
Weight (kg)	79.5 (± 16.2)	78.8 (± 15.2)	80.3 (± 17.3)	0.360
Height (cm)	167.2 (± 9.1)	168.2 (± 7.2)	166.0 (± 10.8)	0.020
BMI (kg/m^2^)	28.9 (± 12.7)	27.9 (± 5.3)	30.1 (± 17.4)	0.093
Diabetes	40 (9.9%)	20 (9.5%)	20 (10.4%)	0.778
Hypertension	121 (30.0%)	59 (28.1%)	62 (32.1%)	0.378
Chronic alcoholism	30 (7.4%)	17 (8.1%)	13 (6.7%)	0.603
Tobacco consumption	8 (2.0%)	6 (2.9%)	2 (1.0%)	0.3
Cognitive impairment	207 (51.4%)	89 (42.4%)	118 (61.1%)	< 0.001
ADL score	6.0 (6.0, 6.0)	6.0 (6.0, 6.0)	6.0 (6.0, 6.0)	0.4
IADL score	4.0 (4.0, 4.0)	4.0 (4.0, 4.0)	4.0 (4.0, 4.0)	< 0.001
Total CES-D	10.0 (8.0, 12.0)	10.0 (10.0, 15.0)	10.0 (8.0, 12.0)	< 0.001
Frailty (SOF scale)	144 (35.7%)	16 (7.6%)	128 (66.3%)	< 0.001
Self-reported VI	356 (88.3%)	176 (83.8%)	180 (93.3%)	0.003
Adapted correction of VI (n = 356)	275 (77.2%)	141 (80.1%)	134 (74.4%)	0.202
Hearing impairment	104 (25.8%)	46 (21.9%)	58 (30.1%)	0.062
Having hearing aids (n = 104)	6 (5.8%)	3 (6.5%)	3 (5.3%)	0.9
Falls	21 (5.2%)	5 (2.4%)	16 (8.3%)	0.007
Risk of undernutrition	30 (7.4%)	16 (7.6%)	14 (7.3%)	0.889

*BMI* body mass index, *ADL* activities of daily living, *IADL* instrumental activities of daily living; *SPPB* short physical performance battery, *CES-D* Center for Epidemiologic Studies- Depression, *SOF* study of osteoporotic fractures.

^a^n (%); mean (± SD); median (IQR: Q1, Q3).

^b^Pearson's Chi-squared test; t-Student test; Fisher's exact test; Wilcoxon rank sum test;

### Characteristics of the population according to the SOF Index

Age, sex, marital status, education, cognitive impairment, IADL score, VI and falls were significantly different across frailty groups (Table S2). Furthermore, participants who were professionally active were more likely to present frailty (48.6% vs. 35.9%).

In the full multivariable model, the odds of having possible sarcopenia significantly increased by 2.66 (95% CI 1.29–5.48) and 2.12 (95% CI 1.05–4.3) respectively in participants who self-reported VI and in participants with corrected VI compared with those who did not have VI (Table 2). The score of the chair test decreased among participants with VI compared to those without VI (β = − 0.45 (95% CI − 0.82, − 0.07)). The balance test and the gait speed test were not associated with VI (Table 2). The odds of presenting physical frailty significantly increased by 2.92 (95% CI 1.2–6.87) in participants who self-reported VI compared with those who did not.

**Table 2:** Association between self-reported visual impairment and sarcopenia and the SPPB subtests.

	Sarcopenia		Sub-tests					
			Standing balance test		Gait speed test		5 chair stand test	
	OR (95%CI)	p value	β (95% CI)	p value	β (95% CI)	p value	β (95% CI)	p value
**Short model**								
No VI	1	–	1	–	1	–	1	–
VI (yes)	2.34 (1.17 ; 4.69)	0.012	− 0.13 (− 0.36 ; 0.1)	0.272	− 0.12 (− 0.35; 0.12)	0.342	− 0.44 (− 0.81 ; − 0.07)	0.021
Corrected VI (yes)	2.12 (1.05; 4.3)	0.032	− 0.17 (− 0.41; 0.07)	0.171	− 0.1 (− 0.33; 0.14)	0.425	− 0.36 (− 0.74; 0.02)	0.062
**Full adjusted model**								
No VI	1	–	1	–	1	–	1	–
VI (yes)	2.66 (1.29; 5.48)	0.006	− 0.16 (− 0.39; 0.08)	0.184	− 0.12 (− 0.36; 0.12)	0.327	− 0.45 (− 0.82; − 0.07)	0.019
Corrected VI (yes)	2.47 (1.18; 5.17)	0.013	− 0.21 (− 0.45; 0.03)	0.082	− 0.12 (− 0.36; 0.12)	0.344	− 0.4 (− 0.79; − 0.02)	0.039

Short model was adjusted for age and sex. *aOR* adjusted odds ratio.

^H^Adjusted for: age, sex, marital status, school level, cognitive impairment, IADL score, nutritional status, hypertension and diabetes.

## Discussion

The objective of this study was to investigate the relationship between VI and sarcopenia in an urban population in Cameroon. After adjusting for several factors, VI was significantly associated with sarcopenia (OR 2.66 [95% CI 1.29–5.48]).

The prevalence of VI (88.3%) in this study was high. It is known that this type of measurement (“self-reporting”) is subject to some bias, especially on the part of the respondent, as participants often tend to exaggerate the symptoms. This is particularly true in the African context. Indeed, after our analyses, we approached some Cameroonian ophthalmologists to try to explain this high prevalence and most of the ophthalmologists in the field told us that socio-culturally people tend to say they have a visual disorder when asked during surveys. They hope to receive free examination and treatment, which they do not always have access to; especially those with low levels of education. It is therefore possible that the prevalence of VI is somewhat overestimated. However, it is known that compared to populations in Europe, for example, the prevalence of VT is much higher in sub-Saharan Africa^19,20,22^ or in other low and middle income countries such as Brazil^23^. This is due to the social, cultural and economic context. Therefore, our result is not exceptional and shows the need to pay special attention to vision problems in this population.

In literature, the only study that addressed the relationship between VI and sarcopenia in LMIC reported the increased risk of sarcopenia with the severity of VI^21^. No specific results were provided for the two SSA countries included^21^. The current study is the first to provide results on the association between VI and sarcopenia in a SSA population. We found that self-reported VI was independently associated with sarcopenia in our population despite accounting for undernutrition (one of VI consequences and sarcopenia causes^10^). The underlying mechanisms that may explain the relationship between VI and quality of physical function are not clear^15^. We believe that this association is due to the fact that people with vision impairments are generally less mobile than those without impairments. Indeed, people with VI are slower and therefore less physically active. For example, Gonzales-Turin et al. reported in a longitudinal study that the areas of frailty domains whose appearance was most increased by VI were slowness, low energy, low physical activity and weakness^3^. Similarly, the social isolation of these people would also lead to a reduction in physical activity, as has already been mentioned^17^, but this social isolation was not investigated in our study. Whatever the cause, lack of physical activity could lead to a decrease in muscle strength, putting the person at greater risk of sarcopenia. Some authors believe that VI is associated with cardiovascular risk factors such as diabetes or hypertension which are on their own associated with physical deficiencies^24^. However, the association between VI and sarcopenia remained significant after adjustment for these conditions in our population.

In most SSA countries, there are no institutions for dependent older people, and they represent an important daily burden for their families. In addition, in clinic field, VI could be an early indicator or warning sign for physical impairments. As vision disorders are most often due to refractive errors, Lee et al. suggest that correction of refractive errors could prevent or delay the process of disruption involved in frailty^25^. This evidence seems to suggest that it would be important to carry out public health actions for early detection and management of refractive errors in the older people to reduce their risk to present physical impairment or to progress to dependence. Nevertheless, these associations should be consolidated by futures studies (if possible longitudinal) in this population. Even when corrected, VI remained associated with sarcopenia. This may be due to two hypotheses: (1) the correction may not have been appropriate and (2) the correction may have come after the onset of sarcopenia. Future longitudinal studies should evaluate the effect of correcting VI before the onset of sarcopenia and make public health recommendations.

As seen earlier, VI was associated with SPPB in our sample. Among the sub-tests of the SPPB, only the chair lift test was associated with VI. This result might be surprising given that eye opening has a direct role in balance, therefore, one would expect visual disorders to be particularly related to the balance test. A previous study showed that although all three tests of the SPPB contributed significantly to the total score, the contribution of the balance test was greater than that of the others^26^. But this population was older than ours (mean 86.5 vs. 67.1 years). We could assume that, on the one hand, the contribution of these sub-tests to the total score could be a function of age. On the other hand, for younger people, a low chair lift score may be better marker of physical performance and therefore more associated with VI. One might also expect the walking speed test to be associated with VI as suggested by some authors^14^ by direct effect of VI on walking ability. This non-association should be investigated and confirmed by cohort studies, but in general, the possibility that the mechanisms by which VI influence physical performance may change according to age.

The relationship between VI and physical frailty has been investigated in different populations^3,4,7–9^. In most studies, people with VI had an increased risk of frailty compared to those without VI, irrespective of whether VI was objectively assessed^14,25^ or self-reported^3,17^, and whether studies were cross-sectional^14,17,25^ or longitudinal^3,14,17^. The only study that has explored the relationship between VI and physical frailty in an SSA population is from Gbessemehlan et al.^19^. In this study, self-reported VI was associated with frailty (adjusted odds ratio = 2.2; 95% CI 1.1–4.3) but not with pre-frailty (adjusted odds ratio = 1.8; 95% CI 0.9–3.7). Participants in this study were more likely to present frailty due to their lower cognitive performance and their advanced age (median age 75 years). In our study, VI was also associated with physical frailty. This observation in our sample suggests that the association between visual impairment and frailty is not restricted to the very old population^3,17–19^. If visual impairment is shown in further studies to be a risk factor for frailty in this population, it would therefore be important to manage it as early as possible in order to delay the risk of progressing to dependency.

This is the first study in Cameroon that investigated the relationship between VI and frailty and the first in SSA that investigated the relationship between VI and sarcopenia. Moreover, the quality of the data is reliable because the data were collected by clinicians trained in geriatric assessment. In addition, we achieved a good completeness of the data, which maximized the statistical power of sound analyses. Another strength of this work was the important number of variables that were collected. This allowed us to account for numerous potential confounding factors, although all psycho-social factors that can influence the association were not assessed.

Despite these strengths, our study has some limitations. The first was it cross-sectional design. This does not allow to assess the sequence of happening between the onset of VI and sarcopenia/frailty. Nevertheless, some cohort studies have assessed the longitudinal relationship between VI and frailty and the results were similar^14,17^. The participants of the current study were recruited from the subscribers of an insurance company, which could limit the representativeness of the sample. Self-reporting of VI may have created a non-differential bias, since it is assumed that it is not different depending on whether one is fragile or not; the problem being mainly socio-cultural. We cannot therefore determine in which direction this limitation might have impacted the relationship between VI and frailty/sarcopenia. Indeed, it may have increased or decreased the measure of association. However, it has been shown that the prevalence obtained by self-reporting is highly correlated with that obtained by objective measurement. Although this measure is somewhat subjective, it is often used^3,17,19^ and the association with frailty remains similar whether the measurement is objective or not^18^. Furthermore, the fact that 68.2% (275/403) of our population (or 77.2% of all those who reported having visual disorders; see Table 1 in the manuscript) wore corrective glasses is proof that the prevalence of visual disorders is high in this population and that this high prevalence is not only related to a measurement bias.

In conclusion, VI was associated with physical impairment in a geriatric population in SSA. The association between VI has already been studied in other populations in longitudinal studies making VI a risk factor for physical impairments. Longitudinal studies should also be conducted in our population to confirm this risk association and assist the formulation of public health recommendations. Indeed, if this association is confirmed in a longitudinal study in Cameroon, it would mean that screening and treatment of visual impairment could be an early means of combating frailty. 

## Supplementary Information

Supplementary Information.

Supplementary Tables.

## Data Availability

The datasets used and/or analysed during the current study available from the corresponding author on reasonable request.
