# Supplementary material for: Self-reported visual impairment and sarcopenia among older people in Cameroon
Source: Sci Rep. 2022 Oct 21;12:17694. doi: 10.1038/s41598-022-22563-9 (PMC9586958; doi:10.1038/s41598-022-22563-9)
Supplement: Supplementary file 1 — Supplementary Information. [file 41598_2022_22563_MOESM1_ESM.pdf]

Appendix

Appendix 1: SPPB Scale

The SPPB consists of three tests: a hierarchical assessment of standing balance, a short walk at the usual pace of older people, and the transition from sitting in a chair to standing five times [33]. For all tests, a score of zero if they were unable to do the test.

- **Balance test**

Implementation: Participants were asked to stand with their feet as close together as possible, then in a semi-tandem position (the side of the heel of one foot touches the big toe of the other foot for 10 seconds) and finally in a tandem position (the heel of one foot completely in front and touching the toes of the other foot for about 10 seconds).

Scores: The scores were assigned as follows:

- a score of 1 if they were able to stand side by side for 10 seconds, but unable to hold a semi-tandem position for 10 seconds,
- a score of 2 if they were able to hold a semi-tandem position for 10 seconds but unable to hold a full tandem position for more than 2 seconds,
- a score of 3 if they managed to stay in full tandem position for 3 to 9 seconds and
- a score of 4 if they could hold a full tandem position for 10 seconds.

- **Gait speed test**

Implementation: participants were asked to walk four meters at a normal pace and the time required to cover this distance was measured.

Scores: The scores were assigned as follows:

- a score of 1 for a duration > 8.7seconds;
- a score of 2 for a duration ranging from 6.21 to 8.7 seconds;
- a score of 3 for a duration ranging from 4.82 to 6.20 seconds; and
- a score of 4 for a duration < 4.82 seconds.

- **5-chair lift test**

Implementation: Participants were asked to stand up and sit down five times as fast as possible on a chair with their arms crossed over their chest.

Scores: The scores were assigned as follows:

- a score of 1 for a duration >16.7 seconds;
- a score of 2 for a duration ranging from 13.7 to 16.6 seconds;
- a score of 3 for a duration ranging from 11.2 to 13.6 seconds; and
- a score of 4 for a duration <11.1 seconds.

Appendix 2: SOF index scale

Frailty as defined by the SOF index has been identified by the presence of two or more of the following three components:

Loss ≥5% of body weight (regardless of intention to lose weight).

Inability to get up from a chair five times without using the arms, and

Reduction in energy level identified by the answer "no" to the question "Do you feel energetic?" on the geriatric depression scale.

The response to each component was scored, 1 if Yes and 0 if No [34], so the higher the score, the more fragile the patient.
