# Supplementary material for: Self-reported visual impairment and sarcopenia among older people in Cameroon
Source: Sci Rep. 2022 Oct 21;12:17694. doi: 10.1038/s41598-022-22563-9 (PMC9586958; doi:10.1038/s41598-022-22563-9)
Supplement: Supplementary file 2 — Supplementary Tables. [file 41598_2022_22563_MOESM2_ESM.docx]

**SUPPLEMENTAL MATERIALS**

**Table S1**: Sociodemographic and clinical characteristics of women, Douala - Cameroon, 2019.

| **Characteristics** | **Overall**, **N = 200***^1,2^* | **[55,65[**, **N = 75***^1 ,2^* | **[65,75[**, **N = 103***^1,2^* | **[75,88]**, **N=22***^1,2^* |
| --- | --- | --- | --- | --- |
| Age (years) | 66.7 (±6.3) | 60.4 (±2.7) | 68.8 (±2.8) | 78.0 (±3.5) |
| Marital status (Lives alone) | 148 (74.0%) | 48 (64.0%) | 79 (76.7%) | 21 (95.5%) |
| Scolarity |  |  |  |  |
| None/primary | 81 (40.5%) | 27 (36.0%) | 42 (40.8%) | 12 (54.5%) |
| Secondary | 107 (53.5%) | 40 (53.3%) | 58 (56.3%) | 9 (40.9%) |
| Higher | 12 (6.0%) | 8 (10.7%) | 3 (2.9%) | 1 (4.5%) |
| Being professionnally active | 112 (56.0%) | 47 (62.7%) | 54 (52.4%) | 11 (50.0%) |
| Weight (Kg) | 79.9 (±16.1) | 83.8 (±14.8) | 78.6 (±16.8) | 72.9 (±13.9) |
| Height (cm) | 163.2 (±9.8) | 163.4 (±13.6) | 163.5 (±6.6) | 161.5 (±6.6) |
| BMI (kg/m^2^) | 30.9 (±17.0) | 33.9 (±26.6) | 29.4 (±6.1) | 27.8 (±4.4) |
| Diabetes | 18 (9.0%) | 3 (4.0%) | 12 (11.7%) | 3 (13.6%) |
| Hypertension | 72 (36.0%) | 24 (32.0%) | 38 (36.9%) | 10 (45.5%) |
| Chronic alcoholism | 6 (3.0%) | 2 (2.7%) | 4 (3.9%) | 0 (0.0%) |
| Tobacco consumption | 0 (0.0%) | 0 (0.0%) | 0 (0.0%) | 0 (0.0%) |
| Cognitive impairment | 119 (59.5%) | 33 (44.0%) | 65 (63.1%) | 21 (95.5%) |
| ADL score | 6.0 (6.0, 6.0) | 6.0 (6.0, 6.0) | 6.0 (6.0, 6.0) | 6.0 (6.0, 6.0) |
| IADL score | 4.0 (4.0, 4.0) | 4.0 (4.0, 4.0) | 4.0 (4.0, 4.0) | 4.0 (3.2, 4.0) |
| Balance test score | 4.0 (3.0, 4.0) | 4.0 (3.0, 4.0) | 4.0 (3.0, 4.0) | 3.0 (2.0, 4.0) |
| Gait test score | 3.0 (2.0, 4.0) | 3.0 (3.0, 4.0) | 3.0 (2.0, 3.0) | 3.0 (2.0, 3.0) |
| Five times sit-to-stand | 3.0 (1.0, 3.0) | 3.0 (2.0, 4.0) | 3.0 (1.0, 3.0) | 2.0 (1.0, 2.0) |
| Possible sarcopenia (SPPB scale) | 116 (58.0%) | 41 (54.7%) | 59 (57.3%) | 16 (72.7%) |
| Total CES-D | 10.0 (9.0, 15.0) | 10.0 (8.0, 13.0) | 10.0 (10.0, 16.0) | 12.0 (10.0, 15.2) |
| Frailty (SOF scale) | 92 (46.0%) | 29 (38.7%) | 45 (43.7%) | 18 (81.8%) |
| Self-reported Vision impairment (VI) | 185 (92.5%) | 71 (94.7%) | 95 (92.2%) | 19 (86.4%) |
| Adapted correction of VI (n=356) | 152 (82.2%) | 61 (85.9%) | 76 (80.0%) | 15 (78.9%) |
| Hearing impairment | 54 (27.0%) | 17 (22.7%) | 28 (27.2%) | 9 (40.9%) |
| Having hearing aids (n=104) | 5 (9.4%) | 0 (0.0%) | 3 (11.1%) | 2 (22.2%) |
| Falls | 20 (10.0%) | 10 (13.3%) | 4 (3.9%) | 6 (27.3%) |
| Undernutrition | 13 (6.5%) | 1 (1.3%) | 11 (10.7%) | 1 (4.5%) |

*^1^*Mean (±SD); *^2^*Median (IQR); n (%); BMI: Body mass index, ADL: Limitations in Activities of daily living; IADL: Limitations in instrumental activities of daily living; SPPB: Short Physical Performance Battery; CES-D: Center for Epidemiologic Studies- Depression; SOF: Study of Osteoporotic Fractures

**Table S1bis**: Sociodemographic and clinical characteristics of men, Douala - Cameroon, 2019.

| **Characteristic** | **Overall**, N = 203*^1,2^* | **[55,65[**, N = 65*^1,2^* | **[65,75[**, N = 106*^1,2^* | **[75,88]**, N = 32*^1,2^* |
| --- | --- | --- | --- | --- |
| Age (years) | 67.6 (±6.2) | 60.8 (±2.5) | 68.8 (±2.9) | 77.4 (±2.4) |
| Marital status (Lives alone) | 28 (13.8%) | 8 (12.3%) | 13 (12.3%) | 7 (21.9%) |
| Scolarity |  |  |  |  |
| None/primary | 44 (21.7%) | 10 (15.4%) | 26 (24.5%) | 8 (25.0%) |
| Secondary | 123 (60.6%) | 38 (58.5%) | 63 (59.4%) | 22 (68.8%) |
| Higher | 36 (17.7%) | 17 (26.2%) | 17 (16.0%) | 2 (6.2%) |
| Being professionnally active | 51 (25.1%) | 20 (30.8%) | 27 (25.5%) | 4 (12.5%) |
| Weight (Kg) | 79.1 (±16.4) | 83.3 (±17.2) | 78.8 (±15.2) | 71.7 (±16.2) |
| Height (cm) | 171.0 (±6.5) | 171.4 (±7.4) | 171.4 (±5.9) | 168.8 (±6.3) |
| BMI (Kg/m^2^) | 27.0 (±5.2) | 28.3 (±5.6) | 26.8 (±4.8) | 25.0 (±5.0) |
| Diabetes | 22 (10.8%) | 8 (12.3%) | 12 (11.3%) | 2 (6.2%) |
| Hypertension | 49 (24.1%) | 13 (20.0%) | 24 (22.6%) | 12 (37.5%) |
| Chronic alcoholism | 24 (11.8%) | 7 (10.8%) | 13 (12.3%) | 4 (12.5%) |
| Tobacco consumption | 8 (3.9%) | 5 (7.7%) | 3 (2.8%) | 0 (0.0%) |
| Cognitive impairment | 88 (43.3%) | 18 (27.7%) | 51 (48.1%) | 19 (59.4%) |
| ADL score | 6.0 (6.0, 6.0) | 6.0 (6.0, 6.0) | 6.0 (6.0, 6.0) | 6.0 (6.0, 6.0) |
| IADL score | 4.0 (4.0, 4.0) | 4.0 (4.0, 4.0) | 4.0 (4.0, 4.0) | 4.0 (4.0, 4.0) |
| Balance test | 4.0 (3.0, 4.0) | 4.0 (4.0, 4.0) | 4.0 (3.0, 4.0) | 3.0 (3.0, 4.0) |
| Gait test score | 3.0 (3.0, 4.0) | 4.0 (3.0, 4.0) | 3.0 (3.0, 4.0) | 3.0 (2.0, 4.0) |
| Five times sit-to-stand | 3.0 (2.0, 4.0) | 3.0 (2.0, 4.0) | 3.0 (2.0, 4.0) | 3.0 (2.0, 4.0) |
| Possible sarcopenia (SPPB scale) | 77 (37.9%) | 19 (29.2%) | 38 (35.8%) | 20 (62.5%) |
| Total CES-D | 10.0 (8.0, 12.0) | 10.0 (8.0, 11.0) | 10.0 (8.0, 11.8) | 10.0 (9.0, 12.0) |
| Frailty (SOF scale) | 52 (25.6%) | 9 (13.8%) | 30 (28.3%) | 13 (40.6%) |
| Self-reported VI | 171 (84.2%) | 51 (78.5%) | 89 (84.0%) | 31 (96.9%) |
| Adapted correction of VI (n=356) | 123 (71.9%) | 39 (76.5%) | 65 (73.0%) | 19 (61.3%) |
| Hearing impairment | 50 (24.6%) | 10 (15.4%) | 29 (27.4%) | 11 (34.4%) |
| Having hearing aids (n=104) | 1 (2.0%) | 0 (0.0%) | 1 (3.6%) | 0 (0.0%) |
| Falls | 1 (0.5%) | 0 (0.0%) | 0 (0.0%) | 1 (3.1%) |
| Undernutrition | 17 (8.4%) | 3 (4.6%) | 9 (8.5%) | 5 (15.6%) |

*^1^*Mean (±SD); *^2^*Median (IQR); n (%); BMI: Body mass index, ADL: Limitations in Activities of daily living; IADL: Limitations in instrumental activities of daily living; SPPB: Short Physical Performance Battery; CES-D: Center for Epidemiologic Studies-Depression; SOF: Study of Osteoporotic Fractures.

**Table S2**: Sociodemographic and clinical characteristics of participants according to frailty, Douala - Cameroon, 2019.

| **Characteristics** | **Overall**, N = 403*^1^* | **Robust**, N = 259*^1^* | **Frail**, N = 144*^1^* | **p-value***^2^* |
| --- | --- | --- | --- | --- |
| **Age (years)** | 67.1 (±6.2) | 66.2 (±5.7) | 68.8 (±6.8) | <0.001 |
| **Sex (Males)** | 203 (50.4%) | 151 (58.3%) | 52 (36.1%) | <0.001 |
| **Marital status (Lives alone)** | 176 (43.7%) | 96 (37.1%) | 80 (55.6%) | <0.001 |
| **Scolarity** |  |  |  | <0.001 |
| None/primary | 125 (31.0%) | 64 (24.7%) | 61 (42.4%) |  |
| Secondary | 230 (57.1%) | 156 (60.2%) | 74 (51.4%) |  |
| Higher | 48 (11.9%) | 39 (15.1%) | 9 (6.2%) |  |
| **Being professionnally active** | 163 (40.4%) | 93 (35.9%) | 70 (48.6%) | 0.013 |
| **Weight (Kg)** | 79.5 (±16.2) | 79.8 (±16.1) | 79.1 (±16.6) | 0.671 |
| **Height (cm)** | 167.2 (±9.1) | 167.8 (±7.5) | 166.1 (±11.5) | 0.109 |
| **BMI (Kg/m^2^)** | 28.9 (±12.7) | 28.4 (±5.7) | 30.0 (±19.8) | 0. 353 |
| **Diabetes** | 40 (9.9%) | 27 (10.4%) | 13 (9.0%) | 0.7 |
| **Hypertension** | 121 (30.0%) | 73 (28.2%) | 48 (33.3%) |  |
| **Chronic alcoholism** | 30 (7.4%) | 21 (8.1%) | 9 (6.2%) |  |
| **Tobacco consumption** | 8 (2.0%) | 6 (2.3%) | 2 (1.4%) |  |
| **Cognitive impairment** | 207 (51.4%) | 115 (44.4%) | 92 (63.9%) | <0.001 |
| **ADL score** | 6.0 (6.0, 6.0) | 6.0 (6.0, 6.0) | 6.0 (6.0, 6.0) | 0.014 |
| **IADL score** | 4.0 (4.0, 4.0) | 4.0 (4.0, 4.0) | 4.0 (4.0, 4.0) | <0.001 |
| **Imbalance test score** | 4.0 (3.0, 4.0) | 4.0 (3.0, 4.0) | 3.0 (3.0, 4.0) | <0.001 |
| **Gait test score** | 3.0 (3.0, 4.0) | 3.0 (3.0, 4.0) | 3.0 (2.0, 3.0) | <0.001 |
| **Five times sit-to-stand** | 3.0 (2.0, 4.0) | 3.0 (3.0, 4.0) | 1.0 (1.0, 2.0) | <0.001 |
| **Possible sarcopenia (SPPB scale)** | 193 (47.9%) | 65 (25.1%) | 128 (88.9%) | <0.001 |
| **Total CES-D** | 10.0 (8.0, 12.0) | 10.0 (8.0, 10.0) | 12.0 (10.0, 17.0) | <0.001 |
| **Self-reported VI** | 356 (88.3%) | 220 (84.9%) | 136 (94.4%) | 0.004 |
| **Adapted correction of VI (n=359)** | 275 (77.2%) | 177 (80.5%) | 98 (72.1%) | 0.066 |
| **Hearing impairment** | 104 (25.8%) | 59 (22.8%) | 45 (31.2%) | 0.063 |
| **Having hearing aids (n=104)** | 6 (5.8%) | 2 (3.4%) | 4 (9.1%) | 0.397 |
| **Falls** | 21 (5.2%) | 1 (0.4%) | 20 (13.9%) | <0.001 |
| **Undernutrition** | 30 (7.4%) | 17 (6.6%) | 13 (9.0%) | 0.4 |

*^1^*n (%); Mean (±SD); Median (IQR)

*^2^*Pearson's Chi-squared test; t-Student test; Fisher's exact test; Wilcoxon rank sum test;

BMI: Body mass index, ADL: Activities of daily living; IADL: instrumental activities of daily living; SPPB: Short Physical Performance Battery; CES-D: Center for Epidemiologic Studies- Depression; SO
